# Incidence and characterization of acute pulmonary embolism in patients with SARS-CoV-2 pneumonia: A multicenter Italian experience

**DOI:** 10.1371/journal.pone.0245565

**Published:** 2021-01-22

**Authors:** Marco Loffi, Valentina Regazzoni, Marco Toselli, Alberto Cereda, Anna Palmisano, Davide Vignale, Francesco Moroni, Gianluca Pontone, Daniele Andreini, Elisabetta Maria Mancini, Alberto Monello, Gianmarco Iannopollo, Gianni Casella, Francesco Monetti, Lorenzo Monti, Giuseppe Ferrillo, Gaetano Liccardo, Elisabetta Tonet, Ottavio Zucchetti, Alberto Cossu, Marco Dugo, Gianluigi Patelli, Pietro Sergio, Antonio Esposito, Antonio Colombo, Francesco Giannini, Raffaele Piccolo, Gian Battista Danzi

**Affiliations:** 1 Department of Cardiology, Ospedale di Cremona, Cremona, Italy; 2 Department of Cardiology, GVM Care & Research Maria Cecilia Hospital, Cotignola (RA), Italy; 3 Experimental Imaging Center, IRCCS San Raffaele Scientific Institute, Milano, Italy; 4 School of Medicine, Vita-Salute San Raffaele University, Milano, Italy; 5 Department of Cardiology, IRCCS Ospedale San Raffaele, Milano, Italy; 6 Department of Cardiology, IRCCS Centro Cardiologico Monzino, Milano, Italy; 7 Department of Cardiology, Guglielmo da Saliceto Hospital, Piacenza, Italy; 8 Department of Cardiology, Ospedale Maggiore, Bologna, Italy; 9 Department of Cardiology, IRCCS Humanitas Clinical and Research Hospital, Rozzano (MI), Italy; 10 Department of Cardiology, Azienda Ospedaliero-Universitaria di Ferrara, Cona (FE), Italy; 11 Department of Cardiology, Ospedale Bolognini, Seriate (BG), Italy; 12 Department of Radiology, Ospedale di Cremona, Cremona, Italy; 13 Department of Cardiology, University of Naples Federico II, Naples, Italy; Institut d'Investigacions Biomediques de Barcelona, SPAIN

## Abstract

**Background and aims:**

Several studies reported a high incidence of pulmonary embolism (PE) among patients with severe acute respiratory syndrome coronavirus-2 (SARS-CoV-2) infection, but detailed data about clinical characteristics, risk factors of these patients and prognostic role of PE are still lacking. We aim to evaluate the occurrence of pulmonary embolism among patients with SARS-CoV-2 infection, and to describe their risk factors, clinical characteristics, and in-hospital clinical outcomes.

**Methods:**

This is a multicenter Italian study including 333 consecutive SARS-CoV-2 patients admitted to seven hospitals from February 22 to May 15, 2020. All the patients underwent computed tomography pulmonary angiography (CTPA) for PE detection. In particular, CTPA was performed in case of inadequate response to high-flow oxygen therapy (Fi0_2_≥0.4 to maintain Sp02≥92%), elevated D-dimer (>0.5μg/mL), or echocardiographic signs of right ventricular dysfunction. Clinical, laboratory and radiological data were also analyzed.

**Results:**

Among 333 patients with laboratory confirmed SARS-CoV-2 pneumonia and undergoing CTPA, PE was detected in 109 (33%) cases. At CTPA, subsegmental, segmental, lobar and central thrombi were detected in 31 (29%), 50 (46%), 20 (18%) and 8 (7%) cases, respectively. In-hospital death occurred in 29 (27%) patients in the PE-group and in 47 (21%) patients in the non-PE group (p = 0.25). Patients in PE-group had a low rate of traditional risk factors and deep vein thrombosis was detected in 29% of patients undergoing compression ultrasonography. In 71% of cases with documented PE, the thrombotic lesions were located in the correspondence of parenchymal consolidation areas.

**Conclusions:**

Despite a low rate of risk factors for venous thromboembolism, PE is present in about 1 out 3 patients with SARS-CoV-2 pneumonia undergoing CTPA for inadequate response to oxygen therapy, elevated D-dimer level, or echocardiographic signs of right ventricular dysfunction. In most of the cases, the thromboses were located distally in the pulmonary tree and were mainly confined within pneumonia areas.

## Introduction

The first case of pneumonia by severe acute respiratory syndrome coronavirus-2 (SARS-CoV-2) was reported in Italy on February 21, 2020 and the northern part of the country rapidly became the center of the pandemic.

COVID-19 has a wide spectrum of clinical manifestations from mild disease, characterized by cough, fever and muscle pain to severe progressive pneumonia with acute respiratory distress syndrome (ARDS), multiorgan failure and death [1,2]. Infected subjects are at increased risk of thromboembolism phenomena [3]. Indeed, abnormal coagulation parameters have been described among patients hospitalized with severe COVID-19, including elevated D-dimer and fibrin degradation products (FDP) levels, with a strong correlation with in-hospital mortality [4,5]. Since the first case of severe acute pulmonary embolism (PE) in a SARS-CoV-2 patient without major predisposing factors for deep vein thrombosis was described [6], many other studies confirmed the high incidence of PE during COVID-19 infection. However, a detailed clinical characterization of patients with PE, the D-dimer’s role in predicting embolism, the nature of PE (real embolism or local inflammation process) and the prognostic value remains poorly described [7].

We aimed to report the rate and the distribution of PE in patients with SARS-CoV-2 infection admitted in seven hospitals located in Northern Italy during the first outbreak of the disease. We also described the risk factors, clinical characteristics and in-hospital outcome of this population.

## Materials and methods

### Study design and patients

This is a retrospective, multicenter, observational, cohort study. The cohort included 333 consecutive patients with a confirmed diagnosis of pneumonia by SARS-CoV-2 admitted to seven hospitals located in Northern Italy between February 22 to May 15, 2020, who underwent computed tomography pulmonary angiography (CTPA) at admission.

Patients were included if they had the following two criteria: a confirmed clinical and radiological diagnosis of SARS-CoV-2 pneumonia, according to the criteria proposed by Chinese Clinical Guidance for COVID-19 Pneumonia Diagnosis and Treatment (7th edition, published online on March 4, 2020), and a positive Reverse-Transcriptase-Polymerase-Chain-Reaction (RT-PCR) assay for SARS-CoV-2 in a respiratory tract sample.

Computed tomography pulmonary angiography was performed by institutional protocol or clinical suspicion of PE according to at least one of the following clinical and laboratory criteria:

- lack of adequate clinical response to high oxygen flow therapy administered for at least 48 hours, defined as need for fraction of inspired oxygen (Fi0_2_)≥0.4 to maintain oxygen saturation (Sp02)≥ 92%;
- high D-dimer levels (>0.5 μg/mL);
- right ventricle dysfunction in terms of systolic pulmonary artery pressure (sPAP) >35 mmHg or tricuspid annular plane systolic excursion (TAPSE) <18 mm evaluated by transthoracic echocardiography.

According to the ESC Guidelines for the management of acute PE [8], simplified Pulmonary Embolism Severity Index (sPESI) was used for risk stratification of patients with diagnosis of PE and without hemodynamic instability [9].

We collected information about medical history, presence of major predisposing risk factors for venous thromboembolism, complete blood chemistry tests, and clinical outcomes, including admission to the Intensive Care Unit (ICU) for mechanical ventilation, death, and discharge at home.

The study was conducted according to the principles expressed in the Declaration of Helsinki. All data were analyzed anonymously and the study was approved by the local ethical committee of Ospedale di Cremona, Cremona, Italy.

### CTPA image acquisition and interpretation

CTPA examinations were performed using a standard CTPA protocol in a multidetector computed tomography (CT) scanner after intravenous injection of 50–75 mL of high concentration iodinated contrast medium [8]. All CTPA scans were interpreted locally by one senior radiologist who was blinded to clinical information.

Four types of PE (subsegmental, segmental, lobar and central) were defined on the basis of the location of the thrombi considering the most proximal pulmonary arterial branch involved.

A semi-quantitative scoring system was used to estimate the relation between lung consolidation and PE localization.

This relationship was classified as follows:

- score 0: all the filling defects out from the ‘‘pneumonia” areas;
- score 1: filling defects mainly out (less than 50% of the emboli) from the ‘‘pneumonia” areas;
- score 2: filling defects mainly inside (at least 50% of the emboli) the ‘‘pneumonia” areas;
- score 3: all the filling defects inside the ‘‘pneumonia” areas.

A semi-quantitative scoring system already validated was used to quantitatively estimate the pulmonary involvement [10].

Briefly, each lung was divided into upper (above the carina), middle, and lower (below the inferior pulmonary vein) zones.

Each zone was evaluated for percentage of lung involvement on a scale of 0 to 4 as follows:

- 0 = 0% involvement,
- 1 = less than 25% involvement,
- 2 = 25% to less than 50% involvement,
- 3 = 50% to less than 75% involvement,
- 4 = 75% or greater involvement.

Overall CT scores were the summation of scores from all six lung zones.

The maximum possible score was 24.

### Statistical analysis

Continuous variables were summarized as median with interquartile range (IQR) and compared with the Mann-Whitney U test. Categorical variables were presented as number (%), and proportions for categorical variables were compared using the χ2 or Fisher exact test as appropriate. Receiver-operating characteristic (ROC) analysis was used to determine the accuracy of quantitative D-dimer measurements in differentiating between positive and negative PE patients. A two-sided p of less than 0.05 was considered statistically significant.

Statistical analyses were done using the SPSS version 21.0 software (IBM, New York, USA).

## Results

A total of 333 patients with confirmed diagnosis of SARS-Cov-2 pneumonia admitted in seven hospitals located in Northern Italy between 22 February 2020 and 15 May 2020 and submitted to CTPA were included in our study. Overall, 63% (211 of 333) were male and the median age was 67 years (IQR 57–77).

The reasons for CTPA assessment were: inadequate clinical response to high oxygen flow therapy (201 patients); D-dimer levels higher than 0.5 μg/mL (184 patients); signs of right ventricle dysfunction at echocardiography (21 patients). A diagnosis of PE was confirmed in 109 cases (33%).

### Baseline characteristics and clinical outcomes

Clinical characteristics of the two groups were similar, although patients with PE had a higher heart rate (HR) and lower systolic blood pressure (SBP) at admission compared with those without PE. No main comorbidities were associated with a higher risk of PE (Table 1). Traditional risk factors for PE such as active cancer, hospitalization or acute coronary syndrome in last 3 months, oral contraceptive therapy, autoimmune diseases, history of previous venous thromboembolism were not associated with PE occurrence (Table 2). Deep vein thrombosis (DVT) was detected in 15 (29% of 51) patients at compression ultrasonography, which was performed in 51 (47%) patients with PE.

**Table 1:** Clinical characteristics of the population.

	Total (n = 333)	PE (n = 109)	Non-PE (n = 224)	p value
**Age (years)**	67 (57–77)	64 (55–76)	67 (57–77)	0.33
**Age > 65 (years)**	175 (53%)	51 (47%)	124 (55%)	0.14
**Gender, Female**	122 (37%)	35 (32%)	87 (39%)	0.23
**Comorbidities**				
*Hypertension*	179 (54%)	57 (52%)	122 (55%)	0.7
*Diabetes*	60 (18%)	17 (16%)	43 (19%)	0.42
*Smoking*	39 (12%)	14 (13%)	25 (11%)	0.65
*Dyslipidemia*	75 (23%)	20 (18%)	55 (24%)	0.2
*Obesity (BMI ≥ 30)*	38 (11%)	16 (15%)	22 (10%)	0.19
*Ischemic heart disease*	44 (13%)	11 (10%)	33 (15%)	0.24
*Cerebrovascular disease*	34 (10%)	9 (8%)	25 (11%)	0.41
*COPD*	27 (8%)	11 (10%)	16 (7%)	0.3
*Atrial fibrillation*	31 (9%)	9 (8%)	22 (10%)	0.65
*Chronic kidney disease*	28 (8%)	8 (7%)	20 (9%)	0.62
**Pa02 at admission**	62 (52–77)	62 (51–74)	63 (54–77)	0.4
**Pa02/Fi02 ratio at admission**	281 (214–333)	274 (202–321)	284 (219–335)	0.52
**Oxygen Saturation at admission**	92 (88–95)	92 (89–95)	92 (88–95)	0.98
**SBP at admission (mmHg)**	125 (115–140)	120 (110–130)	130 (119–140)	**0.001**
**HR at admission (bpm)**	90 (80–103)	94 (80–106)	89 (79–100)	**0.04**
**Pneumonia extension (score)**	13 (5–18)	12 (5–17)	13 (6–19)	0.26

Data are median (IQR) or n (%). PE: pulmonary embolism; COPD: chronic obstructive pulmonary disease; BMI: body mass index; SBP: arterial systolic blood pressure; HR: heart rate; PaO2: arterial oxygen partial pressure; FiO2: fraction of inspired oxygen. Bold numbers indicate significant p-value<0.05.

**Table 2:** Major predisposing risk factors for PE.

	Total (n = 333)	PE (n = 109)	Non-PE (n = 224)	p value
**Hospitalization (last 3 months)**	0	0	0	//
**ACS (last 3 months)**	0	0	0	//
**Previous DVT/PE**	6 (2%)	3 (3%)	3 (1%)	0.4
**Active Cancer**	33 (10%)	8 (7%)	25 (11%)	0.27
**Oral contraceptive therapy**	1 (0.3%)	0	1 (0.5%)	1
**Autoimmune diseases**	11 (3%)	3 (3%)	8 (4%)	1

Data are n (%).

PE: pulmonary embolism; ACS: acute coronary syndrome; DVT: deep vein thrombosis.

Medical therapy before admission (Table 3) included antiplatelet therapy: aspirin or clopidogrel in 56 (17%) patients; 15 (14%) in the PE group and 41 (18%) in the non-PE group; p = 0.5. 9 patients (3%) received anticoagulant therapy with warfarin, 4 (4%) in the PE group and 5 (2%) in the non-PE group (p = 0.48). 17 patients (5%) received anticoagulant therapy with direct-acting oral anticoagulants (DOAC) before admission, 11 (10%) in PE group and 6 (3%) in non-PE group (p = 0.004). 66 (61%) patients in the PE group and 157 (70%) patients in the non-PE group (p = 0.08) received since the admission prophylactic low molecular weight heparin therapy with enoxaparin at daily dosage of 4000 IU; this dosage was increased to 6000 IU in patients with body weight > 80 Kg and halved in those with severe renal impairment (Creatinine Clearance between 15 and 30 ml/min). All 333 patients with PE diagnosis at CTPA received a full anticoagulant dose of enoxaparin (100 IU/Kg twice daily).

**Table 3:** Medications before PE diagnosis.

	Total (n = 333)	PE (n = 109)	Non-PE (n = 224)	p
**Aspirin**	45 (14%)	13 (12%)	32 (15%)	0.55
**Clopidogrel**	11 (3%)	2 (2%)	9 (4%)	0.51
**Warfarin**	9 (3%)	4 (4%)	5 (2%)	0.48
**DOAC**	17 (5%)	11 (10%)	6 (3%)	**0.004**
**LMWH (prophylactic dose)**	223 (67%)	66 (61%)	157 (70%)	0.08
**Hydroxychloroquine**	281 (84%)	88 (81%)	193 (86%)	0.2
**Tocilizumab**	16 (5%)	5 (5%)	11 (5%)	0.9

Data are n (%).

PE: pulmonary embolism; DOAC: direct-acting oral anticoagulants; LMWH: low molecular weight heparin.

None of the patients with PE presented with hemodynamic instability at the time of the diagnosis and therefore thrombolytic therapy was not used in the context of PE. sPESI score was 0 in 35 PE patients (32%); 1 in 36 patients (33%); 2 in 20 patients (18%); 3 in 2 patients (2%) and 4 in 2 patients (2%); complete data to evaluate sPESI score were not available in 14 patients. No PE patient had a sPESI score higher than 4.

No differences were noted between the two study groups about need for non-invasive ventilation with C-PAP (61 [56%] vs 118 [53%], p = 0,57) and ICU admission (29 [27%] vs 50 [22%], p = 0,39). In-hospital death occurred in 29 (27%) patients in the PE-group and in 47 (21%) patients in the non-PE group (p = 0.25).

### Laboratory findings

Laboratory tests at the admission are shown in *Table* 4.

**Table 4:** Laboratory data.

	Normal range	Total (n = 333)	PE (n = 109)	Non-PE (n = 224)	p value
**D-dimer (μg/ml) Admission value**	0–0.5	2.1 (0.6–4.7)	3.6 (0.9–14.7)	1.3 (0.6–3.3)	**0.001**
**D-dimer (μg/ml) Peak value**	0–0.5	3.8 (2.6–9.9)	5.7 (3.3–49)	3.3 (1.9–9.6)	**0.001**
**Hs-TnI (ng/L) Admission value**	0–34	14.7 (8.9–107.8)	13.9 (6–238)	16.7 (8.9–93)	0.69
**Hs-TnI (ng/L) Peak value**	0–34	24.8 (12.8–108.9)	39 (14.6–238)	20.1 (11.2–107.7)	0.85
**CRP (mg/L) Admission value**	0–5	65.5 (22.8–150)	49 (23.4–220)	71 (21–140)	0.34
**CRP (mg/L) Peak value**	0–5	113.5 (48.3–257.7)	99 (33.3–270)	117 (55–210)	0.12
WBC (/mm^3^)	3900–10600	7775 (5115–12837)	14000 (5950–21800)	7340 (4740–11700)	**0.001**
**Hb (g/dL)**	14–18	12.5 (10.9–14.2)	13.9 (12.1–16)	12.2 (10.8–14)	**0.015**
PTL (*10^3^/mm^3^)	150–400	230 (170.7–307.5)	232 (142–330)	228 (175–273)	0.84
**aPTT (seconds)**	25–36	30 (28.2–33.2)	30 (27.7–31.8)	30 (28.3–34)	0.054
**INR**	0.8–1.2	1.15 (1.08–1.30)	1.33 (1.15–1.53)	1.14 (1.06–1.23)	0.64
**LDH (U/L)**	<248	346 (273–448)	293 (227–476)	354 (289–447)	0.25
**Creatinine (mg/dL)**	0.7–1.18	1.07 (0.79–1.33)	1.09 (0.80–1.41)	1.07 (0.76–1.31)	0.94
**EGFR (ml/min/1.73mq)**	> 60	74.7 (50.3–94.5)	73.7 (48.1–96)	75.7 (51.1–92)	0.48
**IL-6 (ng/L)**	95–100	56.5 (21.7–120)	49 (16–126)	58 (24–118)	0.94

Data are median (IQR) or n (%).PE: pulmonary embolism; hs-TnI: high sensitive troponin I; CRP: C-reactive protein; WBC: white blood cell count; PTL: platelets; ALT: alanine aminotransferase; AST: aspartate transaminase; aPTT: activated partial thromboplastin time; INR: international normalized ratio; LDH: lactate dehydrogenase; EGFR: estimated glomerular filtration rate; Pa02: arterial oxygen partial pressure; S02: oxygen saturation; Fi02:fraction of inspired oxygen. Bold numbers indicate significant p-value<0.05.

Both baseline and peak value of D-dimer were higher in PE-group compared to no-PE group (3.6 μg/mL [IQR 0.9–14.7] vs. 1.3 μg/mL [IQR 0.6–3.3], p = 0.001 for baseline level; 5.7 μg/mL [IQR 3.3–49] vs. 3.3 μg/mL [IQR 1.9–9.6], p = 0.001 for peak value). High-sensitivity cardiac troponin I levels were low and not different between the two groups. Patients with PE presented higher leucocyte level (14000/mm^3^ [IQR 5950–21800] vs. 7340/mm^3^ [IQR 4740–11700].

### D-dimer levels and risk of PE

The ROC curve showed an area under the curve of 0.68 (p <0.001) (*S1 Fig; online supplement*). The best threshold value of basal D-dimer was 2.37 μg/ml with a sensitivity of 70% and a specificity of 62% in detecting PE.

### CTPA findings

**Fig 1:**
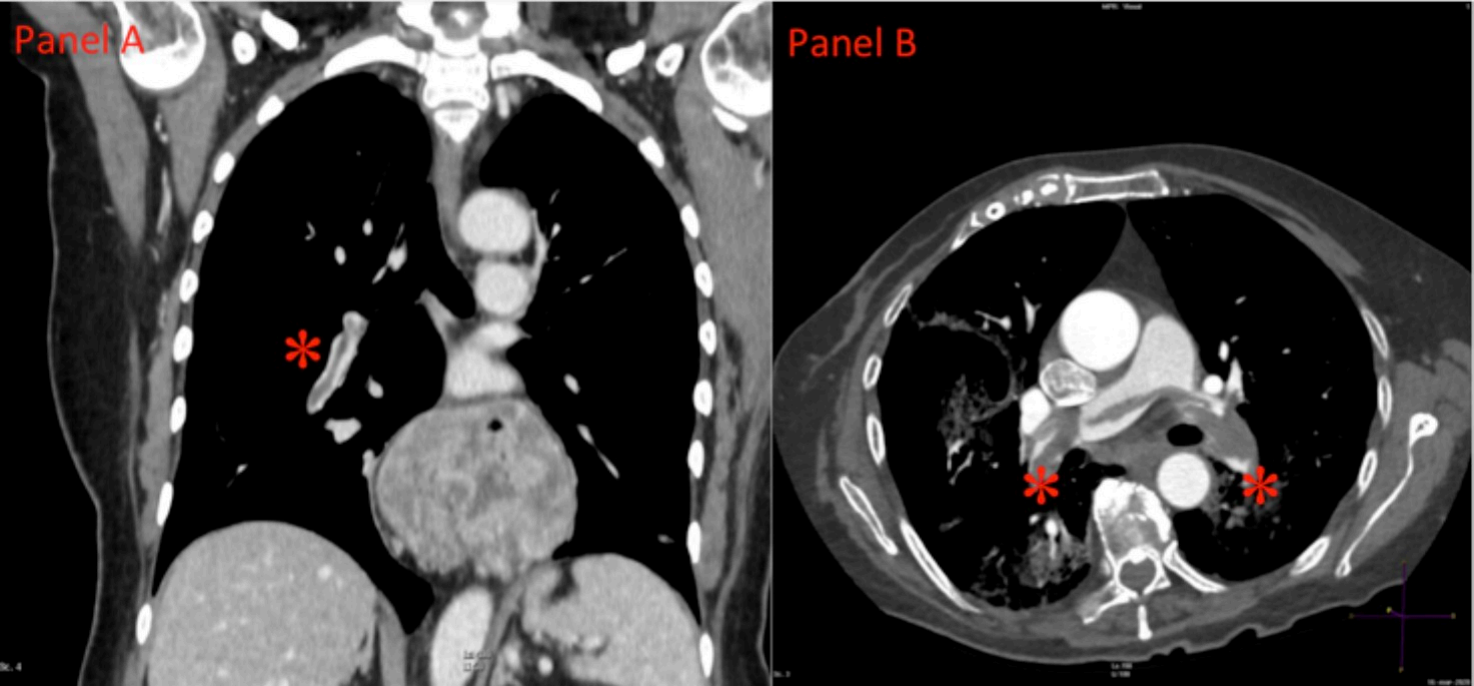
CTPA images of pulmonary embolism. (A) Coronal Computer Tomography Pulmonary Angiography (CTPA) image shows a partial intraluminal filling defect within right interlobar artery (asterisk). (B) Axial CTPA image shows a massive intraluminal filling defect of both main pulmonary arteries (asterisks).

CTPA was performed in all 333 patients and detected PE in 109 (33%). Subsegmental and segmental filling defects were observed in in 31 (29%) and 50 (46%) patients respectively whereas lobar thrombi and central PE were found in 20 (18%) (Fig 1A) and 8 (7%) cases (Fig 1B). Thrombi were bilaterally distributed in 54 (49%) patients.

Pneumonia severity was not different between the two groups as reported in *Table 5*. Chest CT scan acquisition revealed severe pneumonia in 136 (41%) cases with more than 50% of the pulmonary involvement.

**Table 5:** Entity of pulmonary involvement.

	Total (n = 333)	PE (n = 109)	Non-PE (n = 224)	p
**Pulmonary involvement**				
***0%***	37 (11%)	12 (11%)	25 (11%)	0.97
***< 25%***	82 (25%)	29 (27%)	53 (24%)	0.6
***25–50%***	78 (23%)	29 (27%)	49 (22%)	0.34
***50–75%***	82 (25%)	23 (21%)	59 (26%)	0.3
***> 75%***	54 (16%)	16 (14%)	38 (17%)	0.6

Data are n (%).

PE: pulmonary embolism.

**Fig 2:**
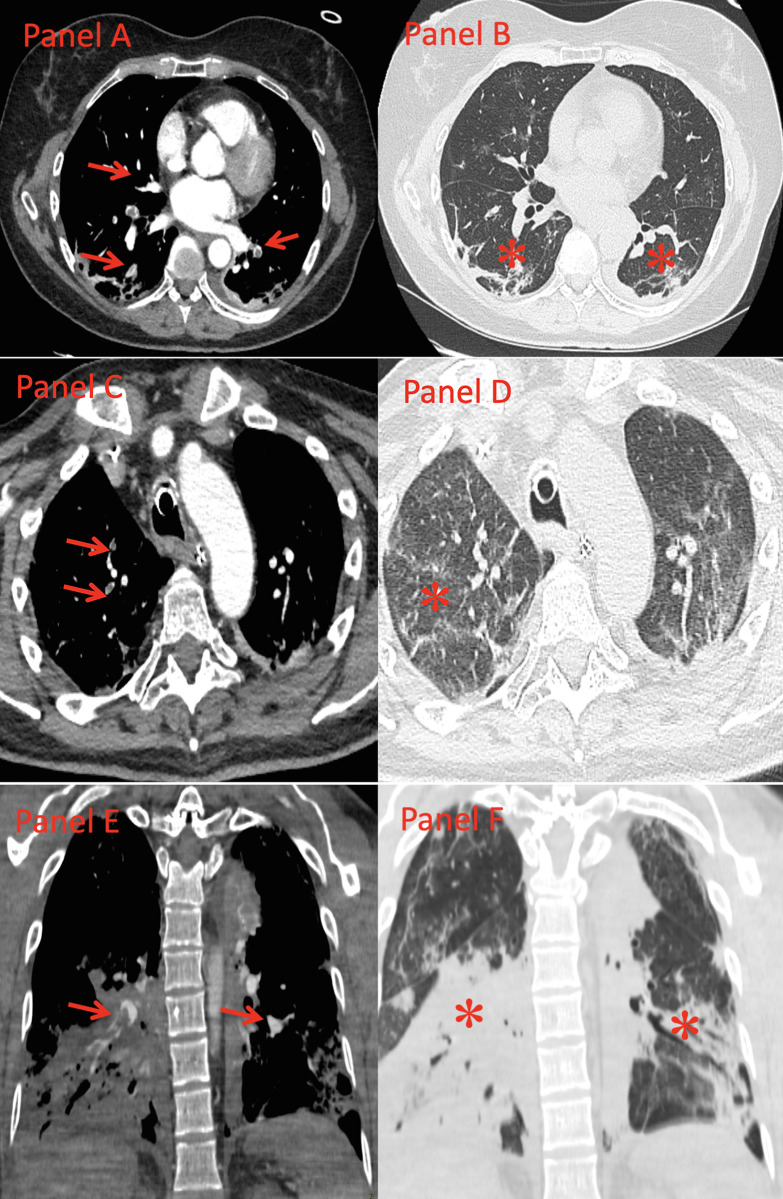
Localization of pulmonary embolism and pneumonia. (A,B) Computed Tomography Pulmonary Angiography shows intraluminal filling defects in some segmental pulmonary arteries of both lower lobes (Panel A, red arrows), localized in lung areas of normal attenuation (Panel B). Subpleural band atelectasis are evident in both lower lobes (Panel B, asterisks). SCORE 0. (C,D) Computed Tomography Pulmonary Angiography shows intraluminal filling defects in two subsegmental pulmonary arteries of the right upper lobe (Panel C, arrows), localized in areas of ground glass attenuation (Panel D, asterisks). A further intraluminal filling defect, not displayed, is present in a segmental pulmonary artery of the right lower lobe. SCORE 2. (E,F) Computed Tomography Pulmonary Angiography shows intraluminal filling defects in some segmental pulmonary arteries of both lower lobes (Panel E, arrows), all localized in areas of lung consolidation (Panel F, asterisks). SCORE 3.

Among PE group, 77 (71%) of the patients CTPA showed PE mainly located in lung consolidation areas (42 cases, 38% with score 3 and 35 cases, 32% with score 2 (Fig 2A–2F). In 32 patients (29%) PE were not primarily located in the pneumonia context (16 cases, 15% with score 1 and 16 cases, 15% with score 0).

## Discussion

In this multicenter study, we reported clinical, radiological characteristics and in-hospital outcomes of a cohort of 333 patients with COVID-19 pneumonia admitted at seven Italian hospitals, who underwent CTPA because of clinical suspicion of pulmonary embolism.

In our cohort PE was present in 33% of cases. This incidence is consistent with data reported in previous case series [11–13] but it is significantly higher in comparison to the rate of 8.3% recently observed in a large French multicenter study [14]. This difference may be due to the fact that the French cohort included a majority of non-severely ill patients; indeed, mortality rate in our patients resulted to be significantly higher (23% vs. 12.2%). Interestingly in both studies, PE was not associated with a higher risk of in-hospital mortality. These findings could be explained by the fact that in both series, PE was located in the distal segments of the pulmonary artery (segmental and subsegmental branches) in 75% of the cases, and therefore, this could have had a limited impact on hemodynamic stability and prognosis. Indeed, in our experience, no one of the patients needed a thrombolytic treatment.

Our results underline the close link between COVID-19 and prothrombotic diathesis. Several reports showed that various infectious conditions are associated with the development of venous thromboembolism (VTE) [15,16] and a substantial portion of patients with PE has an underlying acute infectious condition, especially respiratory tract infections [17,18]. Although the precise mechanism underpinning this association has not yet been clarified, there is evidence suggesting that infections can promote thrombosis through endothelial injury, tissue factor-induced activation of the procoagulant pathway, down-regulation of the endogenous anticoagulant pathway, and inhibition of fibrinolysis [19–22]. Data recently reported by Poissy et al. [23], suggested that local inflammation might play a predominant role in the pathobiological mechanisms of PE in the setting of SARS-CoV-2 pneumonia. Furthermore, recent autopsy studies found vascular thrombosis in lung vessels of patients affected by SARS-CoV-2 pneumonia, suggesting the presence of a localized thrombophylic disorder, related to the infection [24] instead of an embolic phenomenon. Our findings, in terms of lower incidence of deep vein thrombosis, lower rate of traditional risk factors for VTE, associated with the predominant localization of thromboses (71% of cases) in the correspondence of the consolidation areas of the pulmonary parenchyma strongly support this hypothesis.

Interestingly, we observed that patients on chronic anticoagulation therapy with DOAC were not protected from the occurrence of PE and this is in contrast with data recently reported by Fauvel et al. [14]. Our findings might be explained by the fact that DOAC therapy acts only on the coagulation cascade without the additional anti-inflammatory and endothelial-protection properties associated with heparin that could be particularly useful in this specific setting of patients [25,26]. In line with our results, a recent retrospective study by Ameri et al. [27] reported that an oral anticoagulant therapy prior to the admission for COVID-19 tended to be more frequent among patients who eventually suffered from PE, suggesting a lingering pro-thrombotic status despite oral anticoagulant therapy in this subgroup of patients.

However, in this experience, also prophylactic therapy with enoxaparin during hospitalization was not associated with benefits on PE occurrence. In agreement with previous evidences in severely ill COVID-19 patients [11,13,28], our results seem to confirm that patients presenting with a more severe expression of the disease and with a higher thrombogenic status may benefit from an intensive anticoagulation treatment. Furthermore, a therapeutic anticoagulation could lead to a prognostic benefit by preventing the contribution of microvascular thrombosis to the progression of disease [13,29,30]. Based on the results of our study, a risk-adapted approach to escalating the dose of anticoagulation should be considered after assessing the individual thrombotic and bleeding risk in COVID-19 patients: ICU setting, elevated procoagulant proteins or elevated D-dimer could be used to tailor anticoagulation dosages. However, at this moment the optimal dosing in patients with severe COVID-19 remains unknown and warrants further prospective investigation [29].

## Limitations

This study has some limitations. We did not investigate the efficacy of a full dose therapeutic anticoagulant therapy in term of prognostic benefit and bleeding events, so we cannot be conclusive on the best therapeutic choice to start with, since the beginning of the infection. The hypothesis that PE represents a local thrombosis process more than a real embolism related to a more aggressive inflammatory local response, remains speculative. Whether the use of more potent anti-inflammatory drugs among these patients might improve their prognosis remains to be further elucidated. However, the low rate of DVT, the low number of major predisposing thrombo-embolic risk factors and the high number of thromboses located in the pneumonia context let us argue that inflammation and consequent prothrombotic diathesis could play a leading role in PE related to COVID-19. Prophylactic anticoagulation was not used in all subjects. Patients were included in the study during the initial outbreak of COVID-19 in Italy before the WHO recommended the use of routine prophylactic anticoagulation in patients with COVID-19 (March 13^th^, 2020). Finally, we did not perform CPTA routinely to all patients admitted to the hospital with SARS-CoV-2 pneumonia. Although such a study is unlikely to be performed in future, the real incidence of PE among all patients with SARS-CoV-2 pneumonia remains unknown.

## Conclusion

Among patients hospitalized with SARS-CoV-2 pneumonia and clinical suspicious of PE, PE was found in 33% of cases despite a low rate of risk factors for thrombo-embolic events and of DVT. In most of the cases the thromboses were distal and confined in the pneumonia context suggesting a localized coagulopathy more than a classic embolic process. PE was not related with a higher in-hospital mortality. D-dimer levels were strongly associated with PE.

## Supporting information

S1 Fig D dimer levels and risk of PE. ROC curve for basal value of D-Dimer to predict pulmonary embolism. AUC: area under the curve.
(DOCX)

S1 Dataset (XLSX)
