# Supplementary material for: Incidence and characterization of acute pulmonary embolism in patients with SARS-CoV-2 pneumonia: A multicenter Italian experience
Source: PLoS One. 2021 Jan 22;16(1):e0245565. doi: 10.1371/journal.pone.0245565 (PMC7822531; doi:10.1371/journal.pone.0245565)
Supplement: S1 Fig — ROC curve for basal value of D-Dimer to predict pulmonary embolism. AUC: area under the curve. (DOCX) [file pone.0245565.s001.docx]

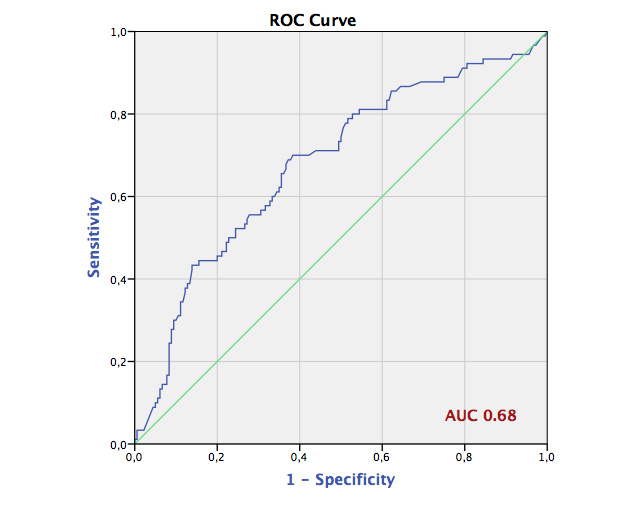
**S1 Fig. D dimer levels and risk of PE**. ROC curve for basal value of D-Dimer to predict pulmonary embolism. AUC: area under the curve.
